# Mode révélateur original et localisation métastatique particulière d’un synovialo-sarcome chez un adulte immunocompétent: à propos d’un cas avec revue de la littérature

**DOI:** 10.11604/pamj.2017.28.103.13200

**Published:** 2017-10-04

**Authors:** Amina Atmane, Sanaa Hammi, Asmâa Regragui, Mohammed Raoufi, Karima Marc, Mouna Soualhi, Rachida Zahraoui, Jouda Benamor, Jamal Eddine Bourkadi

**Affiliations:** 1 Service de Pneumo-phtisiologie, Hôpital Moulay Youssef, Faculté de Médecine et de Pharmacie, Université Mohammed V, CHU Ibn Sina, Rabat, Maroc; 2 Faculté de Médecine et de Pharmacie de Tanger, Université Abdelmalek Essaadi, Tetouan, Maroc; 3 Service Anatomopathologie, Hôpital Universitaire International Cheikh Zaid, Rabat, Maroc

**Keywords:** Synovialo-sarcome, métastase endobronchique, bourgeon bronchique, Synovial sarcoma, endobronchial metastasis, bronchial bud

## Abstract

Le synovialo-sarcome (SS) est une tumeur rare, avec atteinte thoracique rare et de localisation variée. Du fait d’une croissance lente, la tumeur peut être à tort reconnue comme bénigne. Moins de 10% des cas se présente au stade de métastases. Les métastases endobronchiques sont exceptionnelles. L’étude immuno-histochimique et l’analyse cytogénétique permettent de le distinguer des autres tumeurs mésenchymateuses. La présence d’un transcrit de fusion SYT-SSX permet d’affirmer le diagnostic. Le traitement repose sur la chirurgie pour les tumeurs localisées qui peut être associée à la radiothérapie, et sur la chimiothérapie pour les formes métastatiques. Le taux moyen de récidive locorégionale ou métastatique à deux ans des SS est de 50%. Nous rapportons le cas de métastase de SS, exceptionnel par son siège, il s’agit de métastase endobronchique ayant pu révéler la maladie chez un jeune de 28 ans, une maladie évoluant depuis plus de 2 ans. Le SS est redoutable par son évolution lente et insidieuse, son pronostic reste réservé. A travers cette observation nous soulignons la rareté de la localisation et nous insistons sur l’intérêt d’un diagnostic et d’une prise en charge précoces.

## Abstract (en)

Synovial sarcoma (SS) is a rare tumor. It is characterized by various sites of occurrence but rarely involves the chest. The tumor may be wrongly diagnosed as benign due to its slow growth. Less than 10% of patients present with metastatic cancer. Endobronchial metastases are exceptional. Immunohistochemical examination and cytogenetic analysis allow to distinguish it from other mesenchymal tumors. The presence of SYT-SSX fusion transcript allows the diagnosis. Surgery is used for localized tumors that can be treated with radiation therapy while chemiotherapy is used for metastatic tumors. The average rate of locoregional or metastatic recurrence two years after SS is 50%. We report the case of a 28-year old patient with metastatic SS characterized by its uncommon metastatic site. He presented with endobronchial metastasis revealing his disease, that had evolved for more than 2 years. The SS is life-threatening due to its slow and insidious growth. Prognosis is guarded. This study aimed to emphasize this atypical site for metastatic synovial sarcoma as well as to insist on the role of early diagnosis and treatment.

## Introduction

Le synovialo-sarcome (SS) est une tumeur rare, avec atteinte thoracique rare et de localisation variée. Du fait d’une croissance lente, la tumeur peut être à tort reconnue comme bénigne. Moins de 10% des cas se présente au stade de métastases. Les métastases endobronchiques sont exceptionnelles. L’étude immuno-histochimique et l’analyse cytogénétique permettent de le distinguer des autres tumeurs mésenchymateuses. La présence d’un transcrit de fusion SYT-SSX permet d’affirmer le diagnostic. Le traitement repose sur la chirurgie pour les tumeurs localisées qui peut être associée à la radiothérapie, et sur la chimiothérapie pour les formes métastatiques. Le taux moyen de récidive locorégionale ou métastatique à deux ans des SS est de 50%.

## Patient et observation

**Figure 1:**
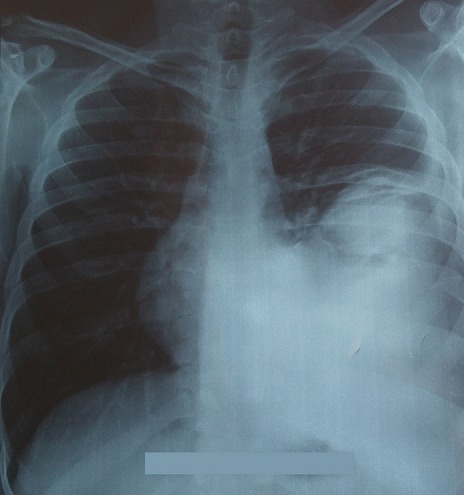
Radiographie thoracique de face: opacité de la moitié inférieure de l’hémithorax gauche dense homogéne

**Figure 2:**
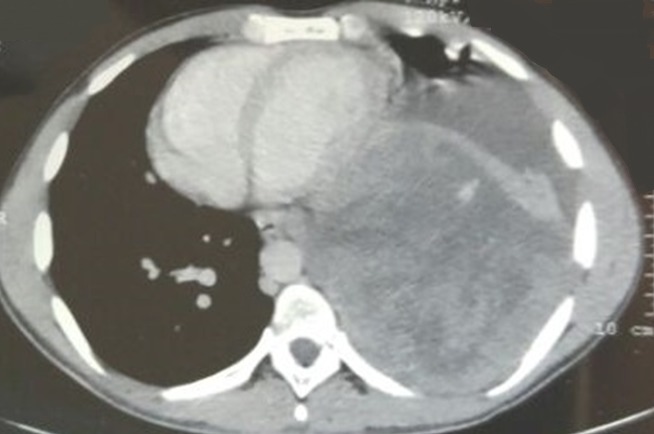
Scanner thoracique: volumineux processus basal gauche refoulant les structures médiastinales vers la droite

**Figure 3:**
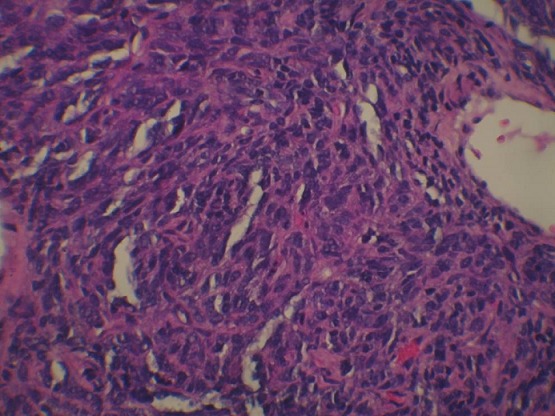
Examen microscopique de la tumeur (Hématéine - éosine x 400): prolifération à cellules fusiformes de densité cellulaire élevée richement vascularisée

**Figure 4:**
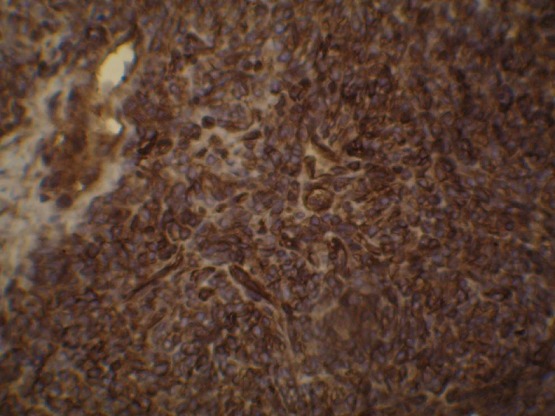
Analyse immuno-histochimique: positivité cytoplasmique diffuse des cellules tumorales par CD99

**Figure 5:**
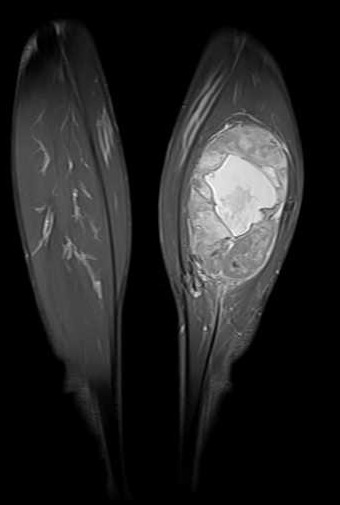
IRM des mollets: masse d’allure sarcomateuse de soléaire gauche

Un jeune patient de 28 ans, sans antécédents pathologiques, accusait des douleurs thoraciques gauches avec une gêne respiratoire, et un amaigrissement chiffré à 8kg en un mois, l’examen clinique objectivait un syndrome de condensation de l’hémi-champs thoracique gauche, avec un mollet gauche légèrement tuméfié, la tuméfaction remontait à 2 ans. La radiographie thoracique objectivait une opacité dense homogène occupant la moitié inférieure de l’hémi-champs thoracique gauche (Figure 1). Le scanner thoracique montrait un volumineux processus basal gauche avec une atélectasie basale et refoulement des structures médiastinales vers la droite (Figure 2). La fibroscopie bronchique objectivait une formation bourgeonnante ayant un caractère obstruant au niveau de l’antéro-basale de la pyramide basale gauche. Les biopsies du bourgeon étaient non contributives. La biopsie transpariétale scanno-guidée de la masse objectivait une prolifération tumorale indifférenciée composée de cellules fusiformes (Figure 3) ou ovoïdes au cytoplasme éosinophile fortement réduit et aux noyaux volumineux à chromatine épaisse avec d’assez nombreuses figures de mitoses. Ces cellules s’agencent en nappes diffuses disposés autour des vaisseaux capillaires ectasiques avec des territoires de nécrose tumorale. Les marqueurs immuno-histochimique était positifs pour les AC anti EMA, anti CD 99 (Figure 4), anti Bcl 2, anti vimentine, anti CD 56, très faiblement positifs pour les AC anti AE1/AE3, et négatifs pour les AC anti Calcrétinine, anti W T 1, anti CD 34 orientant vers un synovialo-sarcome. L’IRM des mollets objectivait une masse d’allure sarcomateuse de soléaire gauche (Figure 5). La biopsie de la masse du mollet confirmait le diagnostic de synovialo-sarcome. Nous avons retenu une localisation thoracique secondaire d’un synovialo-sarcome du mollet.

## Discussion

Le SS est une tumeur rare,de pathogénie inconnue, représentant 7 à 8% des tumeurs malignes d’origine mésenchymateuse. L’âge moyen au moment du diagnostic varie de 26 à 38 ans, avec des observations aux extrêmes de l’âge. Il existe une légère prédominance masculine. Le SS se développe dans 80 à 90% des cas aux dépens des extrémités. Les atteintes thoraciques primitives (8%) sont rares et variées et concernent le cœur, le poumon, le médiastin, l’œsophage, la plèvre et la paroi thoracique, dans 1,5% des cas [1]. Les métastases pulmonaires du SS sont fréquentes et représentent 85%. Cependant les métastases endobronchiques des néoplasies extrathoraciques sont peu fréquentes, de l’ordre de 2% de l’ensemble des métastases. Il s’agit de métastases par voie lymphatique récurrente en sous-muqueux vers la carène et la trachée. A notre connaissance il n’a pas été rapporté dans la littérature de bourgeon métastatique de SS, quelques rares cas de SS endobronchique primitif ont été décrits [2, 3].

La présentation clinique des SS est habituellement celle d´une masse des tissus mous bien limitée, parfois de petite taille notamment dans les localisations tête, cou et mains, non douloureuse et parfois lentement évolutive. Leur localisation est dans 66% des cas située au niveau des extrémités, proche des grosses articulations, mais le site tumoral initial peut être ubiquitaire, bien qu´atteignant plus fréquemment les membres inférieurs. Cette présentation d´allure bénigne est parfois responsable de retards importants au diagnostic avec un délai diagnostique moyen rapporté dans une étude pouvant aller jusqu´à 98 semaines [4], comme le cas de notre patient dont la tuméfaction du mollet étant non douloureuse et ayant survenue après un traumatisme suite à un match de foot ne l’ayant pas motivé à consulter. C’est la symptomatologie pulmonaire faite de douleurs thoraciques et de dyspnée secondaires aux métastases thoraciques qui a pu révéler la maladie.

À l’examen anatomopathologique, la tumeur est de forme ovalaire ou arrondie, parfois multi-nodulaire, souvent bien délimitée et encapsulée, de couleur pâle, blanchâtre ou grisâtre et de consistance molle. On distingue trois sous-types de synovialo-sarcome: la forme monophasique (31%) qui est une forme fibrosarcomateuse pure, la forme biphasique (33%) qui associe des cellules épithéliales et des cellules fusiformes, et la forme indifférenciée (36%) qui contient des cellules de petite taille, de forme ovalaire ou en fuseau caractérisées par un cytoplasme peu abondant et un noyau dense. À l’immunohistochimie, les synovialo-sarcomes expriment dans 90% des cas l’epithelial membrane anti-gen (EMA) et les cytokératines, dans 60% des cas le CD99 et dans 30% des cas la protéine S100. Dans notre cas l’étude immunohistochimique objective la positivité des AC anti EMA, anti CD 99 et anti vimentine. On a retenu une localisation thoracique secondaire devant la présence de la localisation du mollet qui est apparue depuis 2 ans.

La plupart des tumeurs portent une translocation caractéristique t(X; 18), qui implique les gènes SSX1 ou SSX2 du chromosome X (Xp11) et le gène SYT du chromosome 18 (18q11). Les transcrits du gène de fusion SYT-SSX peuvent être détectés sur les prélèvements anatomopathologiques avec une sensibilité de 96% et une spécificité de100% [5]. Des cas familiaux ont été décrits dans la littérature. On n’a pas pu réaliser d’étude génétique chez notre patient par manque de moyens.

En l’absence de sites métastatiques, l’exérèse chirurgicale est le traitement de choix du SS, quelle que soit sa localisation anatomique. De nombreux auteurs ont démontré que le SS est une tumeur chimiosensible. Un traitement par ifosfamide et/ou doxorubicine constitue le traitement de première ligne chez les patients présentant les formes métastatiques, avec un taux de réponse de l’ordre de 50%. Notre patient est décédé avant toute chimiothérapie, témoignant ainsi d’une atteinte sévère. Le taux moyen de récidive locorégionale ou métastatique à deux ans des SS est de 50%.

## Conclusion

Le SS est redoutable par son évolution lente et insidieuse, son pronostic reste réservé. A travers cette observation nous soulignons la rareté de la localisation et nous insistons sur l’intérêt d’un diagnostic et d’une prise en charge précoces.

## Conflits d’intérêts

Les auteurs ne déclarent aucun conflit d’intérêts.
